# MG-HGLNet: A Mixed-Grained Hierarchical Geometric-Semantic Learning Framework with Dynamic Prototypes for Coronary Artery Lesions Assessment

**DOI:** 10.3390/bioengineering13010118

**Published:** 2026-01-20

**Authors:** Xiangxin Wang, Yangfan Chen, Yi Wu, Yujia Zhou, Yang Chen, Qianjin Feng

**Affiliations:** 1 School of Computer Science and Engineering, Southeast University, Nanjing 210096, China; 230208696@seu.edu.cn; 2 School of Biomedical Engineering, Southern Medical University, Guangzhou 510515, China; cyangfan4@gmail.com (Y.C.); yiwu410@126.com (Y.W.); yujia90@smu.edu.cn (Y.Z.)

**Keywords:** coronary artery disease, state space models, weakly supervised learning, deep learning

## Abstract

Automated assessment of coronary artery (CA) lesions via Coronary Computed Tomography Angiography (CCTA) is essential for the diagnosis of coronary artery disease (CAD). However, current deep learning approaches confront several challenges, primarily regarding the modeling of long-range anatomical dependencies, the effective decoupling of plaque texture from stenosis geometry, and the utilization of clinically prevalent mixed-grained annotations. To address these challenges, we propose a novel mixed-grained hierarchical geometric-semantic learning network (MG-HGLNet). Specifically, we introduce a topology-aware dual-stream encoding (TDE) module, which incorporates a bidirectional vessel Mamba (BiV-Mamba) encoder to capture global hemodynamic contexts and rectify spatial distortions inherent in curved planar reformation (CPR). Furthermore, a synergistic spectral–morphological decoupling (SSD) module is designed to disentangle task-specific features; it utilizes frequency-domain analysis to extract plaque spectral fingerprints while employing a texture-guided deformable attention mechanism to refine luminal boundary. To mitigate the scarcity of fine-grained labels, we implement a mixed-grained supervision optimization (MSO) strategy, utilizing anatomy-aware dynamic prototypes and logical consistency constraints to effectively leverage coarse branch-level labels. Extensive experiments on an in-house dataset demonstrate that MG-HGLNet achieves a stenosis grading accuracy of 92.4% and a plaque classification accuracy of 91.5%. The results suggest that our framework not only outperforms state-of-the-art methods but also maintains robust performance under weakly supervised settings, offering a promising solution for label-efficient CAD diagnosis.

## 1. Introduction

Coronary Artery Disease (CAD) remains the leading cause of mortality and disability worldwide, placing a tremendous burden on healthcare systems [1,2]. Coronary Computed Tomography Angiography (CCTA) has emerged as the primary non-invasive modality for CAD diagnosis due to its high spatial resolution and ability to visualize both the coronary artery (CA) lumen and the CA wall [3]. The clinical assessment of CCTA typically involves identifying the presence of atherosclerotic plaque, characterizing its composition (e.g., calcified, non-calcified, mixed), and quantifying the degree of luminal stenosis [4]. These factors are critical for risk stratification and determining the necessity of invasive revascularization.

However, the manual interpretation of CCTA is labor-intensive, time-consuming, and subject to significant inter-observer variability, particularly when assessing plaque characteristics and intermediate stenosis. While expert consensus guidelines like Coronary Artery Disease–Reporting and Data System (CAD-RADS) have standardized reporting [5], the sheer volume of CCTA scans demands automated, accurate, and efficient computer-aided diagnosis systems. While deep learning (DL) has revolutionized medical image analysis [6], existing approaches for CA assessment face three fundamental challenges that hinder their clinical deployment:

- Difficulty in maintaining long-range anatomical and hemodynamic consistency: The physiological significance of a local stenosis is inherently relative, depending on the global context of the continuous, tortuous vessel tree (e.g., proximal plaque burden and distal reference diameter) rather than isolated local features.
- Difficulty in decoupling the plaque texture from stenosis geometry: Plaque characterization relies on spectral signatures (density), while stenosis grading requires morphological boundary delineation. In CCTA, these features are often visually coupled and degraded by artifacts (e.g., calcium blooming), making them hard to distinguish.
- Insufficiency of fine-grained labels and ambiguity of weak supervision: Obtaining fine-grained segment-level labels is labor-intensive, whereas coarse-grained branch-level labels are readily available. However, there is currently a lack of effective strategies to properly utilize these coarse labels to guide fine-grained feature learning, which may lead to negative optimization of the network due to the use of coarse labels.

**Figure 1:**
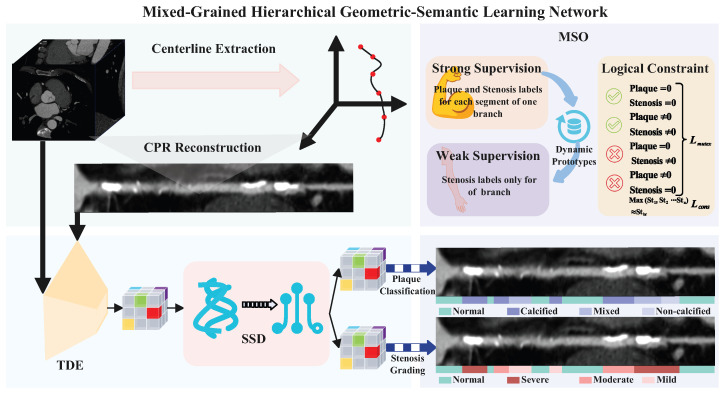
Framework Overview of the proposed MG-HGLNet. The pipeline consists of three core components: (1) Topology-Aware Dual-Stream Encoding (TDE): Captures long-range anatomical dependencies along the vessel tree and rectifies spatial distortions in CPR images. (2) Synergistic Spectral–Morphological Decoupling (SSD): Explicitly disentangles plaque texture from stenosis geometry. (3) Mixed-Grained Supervision Optimization (MSO): Bridges the gap between fine-grained annotations and coarse-grained labels.

To address these challenges, we present a Mixed-Grained Hierarchical Geometric-Semantic Learning Network (MG-HGLNet) for CA stenosis grading and plaque classification (Figure 1). The MG-HGLNet comprises three main components: Topology-Aware Dual-Stream Encoding (TDE), Synergistic Spectral–Morphological Decoupling (SSD) and Mixed-Grained Supervision Optimization (MSO). Specifically, the TDE module is designed to efficiently model the long-range anatomical and hemodynamic dependencies by utilizing a bi-directional vessel Mamba (BiV-Mamba) followed by an attention mechanism to rectify geometric distortions in Curved Planar Reformation (CPR). Then, the SSD module is proposed to decouple stenosis grading and plaque classification tasks. It employs a frequency-band Mamba (FB-Mamba) to extract spectral fingerprints (e.g., calcification energy) via Fast Fourier Transform (FFT) for plaque characterization and simultaneously uses these texture features as a prior to drive a texture-guided deformable boundary attention (TG-DBA), which adaptively shifts the receptive field to capture precise luminal narrowing. Finally, the MSO strategy is presented to overcome the insufficiency of fine-grained annotations. By integrating anatomy-aware dynamic prototypes that align geometric features with diagnoses and logical consistency constraints that penalize branch-segment prediction contradictions, our framework effectively utilizes the coarse-grained labels.

The main contributions of this study are summarized as follows:

- We propose a novel end-to-end MG-HGLNet for CA lesions assessment. Extensive experiments on an in-house dataset demonstrate that MG-HGLNet achieves state-of-the-art performance in both CA lumen stenosis grading and plaque classification.
- We present a TDE module to effectively capture long-range anatomical dependencies and correct spatial distortions inherent in CPR images.
- We design a SSD module that explicitly decouples plaque texture from stenosis geometry by synergizing spectral analysis with deformable morphological attention, significantly enhancing diagnostic interpretability and accuracy.
- We design a dynamic prototype-based learning strategy that bridges the gap between fine-grained segment annotations and coarse-grained clinical reports. Combined with logical mutual exclusion constraints, this allows for efficient utilization of datasets with coarse-grained labels.

## 2. Related Works

### 2.1. Coronary Artery Lesions Assessment

The accurate grading of CA stenosis and the characterization of plaque composition are fundamental to clinical decision-making. This section traces the methodological lineage from traditional algorithms to advanced deep learning (DL)-based methods, delineating the specific mechanisms and limitations inherent to each approach.

Traditional approaches typically rely on simple segmentation like thresholding to detect plaques and handcrafted features followed by classical classifiers such as random forests, support vector machines, or gradient boosting to assess stenosis degree [7,8,9]. However, these methods are often affected by artifacts, and their performance is limited by the quality of handcrafted features and accurate segmentation [10].

Recently, DL-based methods have significantly improved CAD diagnosis. These methods apply deep neural networks (DNNs) to automatically learn hierarchical feature representations from raw images, enabling an end-to-end framework. Convolutional neural network (CNN) is the most common to extract plaque and stenosis features. The majority of methods reconstruct the CA along its centerline, generating multi-planar reformatted (MPR) or CPR images that “straighten” the CA for easier analysis [10,11,12,13,14,15,16,17]. Denzinger et al. [11] proposed a stenosis grading method by analyzing from multi-view of MPR. Penso et al. [18] employed a token-mixer architecture, which can learn structural relationship over the whole CA to analyze the MPR view of a CA segment to diagnose CAD. Surface meshes [14] and 3D clues [19,20] from raw CCTA images along the centerline are also applied to supplement the MPR perspective. Minority methods attempt to segment and analyze plaques directly from raw CCTA images to maintain global anatomical and topological clues. Jiang et al. [21] proposed a CA calcification segmentation model with cross-frequency conditioner for feature disentanglement and geometric prior for reducing false positives. Taking into account the tree-like structure of CA, the estimation of stenosis in a CA segment depends on its context. Therefore, recent methods use architectures capable of modeling long-range dependencies, such as recurrent convolutional neural network (RCNN) [10,22], graph convolutional network (GCN) [12], and Transformers [18,19,23,24]. Although the CA context is effectively captured, the computational and memory requirements increased.

### 2.2. Mamba in Medical Image Analysis

Capturing long-range dependencies is indispensable in medical image analysis, where anatomical structures often span large contexts. While CNN has served as the cornerstone of DL, their intrinsic inductive bias toward local neighborhoods limits their ability to model global semantic interactions. Vision Transformers (ViTs) emerged to alleviate this by leveraging self-attention mechanisms [25]. Recently, to bridge this gap, Gu et al. [26] introduced the Mamba architecture, a Structured State Space Model (SSM) equipped with a Selective Scan Mechanism. This mechanism enabled the model to filter irrelevant data while propagating information across long sequences with linear complexity (O(N)). This paradigm offered a compelling trade-off, combining the global receptive field of Transformers with the inference efficiency of CNNs.

The application of Mamba in medical image processing has become increasingly widespread [27,28,29,30]. Ma et al. [27] proposed U-Mamba, which integrated Mamba blocks into a U-Net encoder. Their hybrid design employed SSM to extract global morphological features while utilizing CNN layers for local texture details. Similarly, Ruan and Xiang [31] developed VM-UNet, a pure SSM-based architecture that replaced standard convolutional layers with Visual State Space (VSS) blocks, achieving superior performance with significantly fewer parameters. For 3D volumetric data, Xing et al. [28] presented SegMamba, which utilized a dimension-aware selective scan mechanism to preserve spatial continuity in volumetric data, effectively addressing the boundary ambiguity in 3D tumor segmentation. Yue and Liu [29] designed a hybrid “SS-Conv-SSM” block that fused the local feature extraction of convolutions with the long-range dependency modeling of SSM.

### 2.3. Weakly Supervised Learning in Medical Imaging

Acquiring pixel-wise expert annotations is a notorious bottleneck in medical imaging due to the high cost and inter-observer variability. Weakly supervised learning addresses this by exploiting coarse or sparse annotations—such as image-level tags, bounding boxes, or scribbles—to achieve dense prediction performance comparable to fully supervised counterparts.

The most challenging weakly supervised learning involves learning solely from image-level labels. The seminal work on class activation maps (CAMs) establishes a baseline by visualizing discriminative regions. However, standard CAMs often suffered from the discrimination-localization trade-off. To mitigate this, Ahn et al. [32] proposed IRNet, which derived displacement fields from inter-pixel affinities to propagate CAM responses to object boundaries. In the medical domain, Huang et al. [33] introduced DeepSEED, a method that employed a seed-based region growing mechanism to iteratively refine the initial CAMs using simple priors. Lu et al. [34] developed CLAM under the multiple instance learning paradigm to enable the localization of tumor sub-regions without pixel-level supervision. Building on this, Shao et al. [35] presented TransMIL, which incorporated Transformer encoders to capture long-range correlations between instances, further improving robustness. A prevailing trend in weakly supervised learning focused on the consistency regularization. For instance, Valvano et al. [36] proposed a multi-scale adversarial learning framework. They employed two discriminator networks to enforce shape consistency between the predictions of the segmentation network and the ground truth scribbles at different scales, effectively propagating label information to unannotated regions while preserving anatomical plausibility.

## 3. Materials and Methods

As illustrated in Figure 1, the MG-HGLNet is proposed for CA lesions assessment, which integrates three core components: (1) a TDE module for feature extraction, (2) an SSD module for decoupling task-specific features and (3) a MSO strategy for effectively utilizing mixed-grained labels. Given a multi-modal input $X=\{X_{MPR},X_{3D},P_{pos}\}$ corresponding to a CA branch, the network predicts 4-class plaque types and 4-class stenosis grading.

### 3.1. Topology-Aware Dual-Stream Encoding

**Figure 2:**
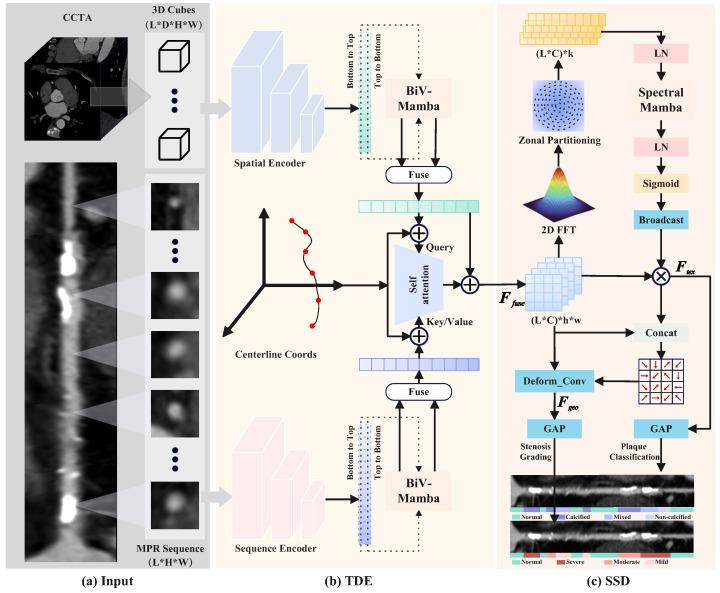
Detailed illustration of the TDE module and SSD module.

To capture the continuous CA structure and rectify geometric distortions inherent in CPR, we construct a dual-stream backbone guided by centerline topology. As shown in Figure 2a, we define the input as a triplet consisting of a sequential stream $X_{MPR}\in R^{L\times H\times W}$ derived from straightened CPR to provide longitudinal textural continuity, a volumetric stream $X_{3D}\in R^{L\times D\times H\times W}$ comprising sliding 3D patches to preserve authentic luminal geometry, and normalized centerline coordinates $P_{pos}\in R^{L\times 3}$ serving as geometric anchors. Initially, both image streams are projected into a high-dimensional feature space via shallow CNNs while explicitly preserving the local spatial resolution (h×w). For the volumetric stream, a depth-wise collapse convolution is applied to encode the 3D anisotropic context into 2D feature maps $e_{vol}\in R^{L\times C\times h\times w}$, ensuring dimensional alignment with the MPR stream feature $e_{mpr}$.

As shown in Figure 2b, to efficiently model the long-range morphological dependencies along the CA with linear computational complexity, we introduce the bidirectional vascular mamba (BiV-Mamba) encoder. Recognizing the anisotropic nature of hemodynamics, we flatten the spatial dimensions of the feature maps into tokens of size $D_{in}=C\cdot h\cdot w$ and apply two independent State Space Models (SSMs) to scan the CA sequence bi-directionally. A linear projection layer expands $D_{in}$ to a latent dimension $D_{model}$ to capture richer contextual representations before applying the SSMs. The forward scan (0→L) follows the physiological direction of blood flow, accumulating proximal anatomical context to establish a baseline CA diameter for identifying downstream narrowings. Conversely, the backward scan (L→0) traverses retrospectively from the distal end, aggregating distal morphological trends to distinguish true pathological stenosis from physiological CA tapering using the patent distal CA caliber as a reference. After sequence modeling, the output tokens are explicitly reconstructed to separate the channel and spatial dimensions, reshaping the tensor back to $R^{L\times C\times h\times w}$. This process yields sequence-optimized content features $H_{seq}$ and $H_{vol}$.

Subsequently, to establish a spatially consistent representation for multi-modal fusion, we inject spatial awareness into the extracted content features. The centerline coordinates $P_{pos}$ are projected via a multi-layer perceptron (MLP) into positional embeddings $PE_{seq}\in R^{L\times C}$. These embeddings are spatially broadcasted across the h×w dimensions to generate the map $PE_{map}$, which is added to the Mamba-encoded features to obtain the topology-aware representations $F_{seq}=H_{seq}+PE_{map}$ and $F_{vol}=H_{vol}+PE_{map}$. While these embeddings contains the implicit long-range correlations, they suffer spatial distortions inherent in the CPR images. Therefore, to enforce local geometric alignment between the straightened MPR sequence and the curved 3D volumetric data, we employ a Spatial-Aware Cross-Attention Module (SAM). We construct a pairwise physical distance matrix $D_{dist}\in R^{L\times L}$ based on the Euclidean distance between centerline coordinates $P_{pos}$, where $D_{i,j}={∥p_{i}-p_{j}∥}_{2}$. This matrix serves as a structural constraint to penalize interactions between spatially distant segments that may share similar textures (e.g., calcifications at different anatomical locations). In the attention mechanism, we utilize the sequential texture feature $F_{seq}$ as the Query (*Q*) to preserve the longitudinal continuity required for plaque analysis, while the volumetric feature $F_{vol}$ serves as the Key (*K*) and Value (*V*) to provide complementary 3D geometric context. The attention scores are computed by incorporating the distance matrix as a subtractive bias term before the Softmax normalization:

(1) $$A_{ij}=\mathrm{Softmax}\left(\frac{Q_{i}K_{j}^{T}}{\sqrt{d_{model}}}-\lambda \cdot \log\left(1+D_{i,j}\right)\right),$$

(2) $$F_{fused}=\mathrm{LayerNorm}\left(F_{seq}+A\cdot V\right),$$

where λ is a learnable scalar controlling the strength of the topological penalty. By fusing features through this geometry-guided attention, TAM effectively performs a geometric calibration, rectifying the distortions in MPR by “looking up” the authentic spatial neighborhood in the 3D volume, resulting in a spatially aligned and geometrically accurate representation $F_{fused}$ for subsequent decoupling.

### 3.2. Synergistic Spectral–Morphological Decoupling

Accurate diagnosis requires disentangling plaque composition, which is texture-dominant, from luminal narrowing, which is geometry-dominant. Therefore, we propose the SSD module (Figure 2c) to achieve this decoupling by exploiting the spectral fingerprints of plaques from the spatially preserved feature maps. Intuitively, plaque characteristics (e.g., calcification, lipid pools) are manifested as high-frequency texture variations, while stenosis is primarily defined by the low-frequency geometric structure of the vessel lumen. Based on this insight, the SSD module employs FB-Mamba to capture frequency-domain discrepancies for plaque classification and TG-DBA to enhance spatial-domain boundary attention for stenosis grading, thereby effectively decoupling these intertwined features.

#### 3.2.1. Spectral-Aware Texture Refinement

For plaque characterization, we introduce the FB-Mamba to capture texture signatures such as the high-frequency energy of calcification versus the low-frequency homogeneity of lipid plaques. We first apply a 2D FFT on the spatial dimensions (h,w) of $F_{fused}$ to obtain the magnitude spectrum. To ensure rotation invariance, we implement a Zonal Partitioning strategy, dividing the spectrum into *K* concentric rings and pooling the energy within each ring to form a spectral sequence $S_{freq}\in R^{L\times C\times K}$. To enhance feature representation capacity, we apply a linear projection layer to map the frequency band dimension *K* to a high-dimensional latent space $d_{model}$. Treating the channel dimension *C* as the sequence length, we then employ a Mamba block to model the inter-channel dependencies based on these embedded spectral distributions, yielding the hidden states $H_{freq}\in R^{L\times C\times d_{model}}$. To translate this spectral context into channel-specific importance scores, we project the latent dimension $d_{model}$ down to a scalar value using a learnable linear weight $W_{p}$ and bias $b_{p}$, followed by a Sigmoid activation σ(·). To enable element-wise calibration with the spatially explicit input feature maps, the resulting channel weight vector is spatially unsqueezed to broadcast across the h×w dimensions. This excitation process is formally formulated as:

(3) $$W_{tex}={\mathrm{Unsqueeze}}_{h,w}\left(\sigma (H_{freq}W_{p}+b_{p})\right),$$

(4) $$F_{tex}=F_{fused}⊙W_{tex},$$

where $W_{tex}\in R^{L\times C\times 1\times 1}$ serves as the modulation gate, effectively suppressing channels dominated by noise while enhancing those containing plaque-specific spectral signatures.

#### 3.2.2. Texture-Guided Morphological Refinement

Simultaneously, for stenosis grading, we propose the Texture-Guided Deformable Boundary Attention (TG-DBA) to extract precise luminal morphology. Recognizing that soft plaques often exhibit blurred boundaries that complicate stenosis estimation, we establish a synergistic bridge where the extracted texture features $F_{tex}$ serve as a conditional prior. We concatenate $F_{fused}$ and $F_{tex}$ to predict pixel-wise sampling offsets $\Delta p\in R^{B\times L\times 2\times h\times w}$ via a convolutional layer. These offsets drive a deformable sampling operation via bilinear interpolation, adaptively shifting the receptive field towards the true CA wall boundaries. Specifically, for each integer spatial location *p* on the feature map grid, the predicted offset yields a fractional coordinate $p^{\prime }=p+\Delta p$. The resampled feature value $F_{geo}\left(p\right)$ is computed by aggregating features from the four-point neighborhood $N(p^{\prime })$, which corresponds to the discrete pixel locations at the top-left, top-right, bottom-left, and bottom-right relative to the fractional point $p^{\prime }$:

(5) $$F_{geo}\left(p\right)=\sum_{q\in N(p^{\prime })}G\left(q,p^{\prime }\right)\cdot F_{fused}\left(q\right),$$

where the bilinear interpolation kernel $G(q,p^{\prime })$ acts as a differentiable proxy defined as $\max(0,1-|q_{x}-p_{x}^{\prime }\left|)\cdot \max(0,1-\right|q_{y}-p_{y}^{\prime }\left|\right)$. This texture-guided geometric feature $F_{geo}$ provides a robust basis for quantifying luminal narrowing.

#### 3.2.3. Task-Specific Diagnostic Projection

To translate the spatially explicit feature maps into segment-level diagnostic probabilities, we employ a dual-head projection architecture. First, we apply Global Average Pooling (GAP) across the spatial dimensions (h,w) of the disentangled features $F_{tex}$ and $F_{geo}$ to aggregate local information into compact feature vectors $v_{tex},v_{geo}\in R^{L\times C}$. Subsequently, these vectors are projected into class-specific probability distributions via task-specific Multi-Layer Perceptrons (MLPs) followed by a Softmax activation. For stenosis grading, the geometry vector $v_{geo}$ is mapped to the 4-class stenosis grading probability distribution $P_{st}\in R^{L\times 4}$:

(6) $$P_{st}=\mathrm{Softmax}\left({\mathrm{MLP}}_{st}\left(v_{geo}\right)\right).$$

Similarly, for plaque characterization, the texture vector $v_{tex}$ is mapped to the 4-class plaque type probabilities $P_{pl}\in R^{L\times 4}$:

(7) $$P_{pl}=\mathrm{Softmax}\left({\mathrm{MLP}}_{pl}\left(v_{tex}\right)\right).$$

Additionally, the fused feature $F_{fused}$ undergoes a parallel global pooling and projection process via an auxiliary head to yield a global branch-level stenosis prediction $P_{global}\in R^{6}$, which serves as a hierarchical reference for consistency constraints.

### 3.3. Mixed-Grained Supervision Optimization

To effectively leverage clinical datasets containing a mix of fine-grained segment annotations and coarse-grained branch diagnoses, we implement a comprehensive optimization strategy driven by dynamic prototypes and logical consistency constraints.

#### 3.3.1. Strong Supervision and Prototype Alignment

For the subset of training data where fine-grained segment-level annotations are available, we optimize the network using strong supervision while simultaneously calibrating the prototype bank to ensure it accurately represents the feature distribution of each stenosis grade. We utilize the standard Cross-Entropy (CE) loss for both the stenosis grading and plaque characterization tasks. Let $y_{st}^{\left(l\right)}$ and $y_{pl}^{\left(l\right)}$ denote the ground-truth labels for the *l*-th segment. The strong supervision loss $L_{strong}$ is calculated as the sum of the losses from both heads:

(8) $$L_{strong}=-\frac{1}{L}\sum_{l=1}^{L}\left[y_{st}^{\left(l\right)}\log\left(P_{st}^{\left(l\right)}\right)+\lambda_{pl}\cdot y_{pl}^{\left(l\right)}\log\left(P_{pl}^{\left(l\right)}\right)\right].$$

These labeled segments serve as the ground truth anchors for constructing the Anatomy-Aware Dynamic Prototypes used in the weakly-supervised branch. During training iterations on strong-label data, we update the prototype vectors $m_{k}$ in the bank $M\in R^{6\times C}$. For each stenosis grade *k*, we compute the mean geometric feature vector ${\bar{v}}_{geo}^{\left(k\right)}$ of all segments in the current batch that belong to class *k*. To initialize the anatomy-aware dynamic prototypes, we employ K-Means clustering (K = 4) on the geometric feature embeddings of the fine-grained labeled data during the warm-up phase. Then, the prototype is then updated via Exponential Moving Average (EMA) to ensure stability: $m_{k}\leftarrow \mu m_{k}+\left(1-\mu \right){\bar{v}}_{geo}^{\left(k\right)}$, where μ is a momentum coefficient and is set to 0.99. This alignment ensures that the prototypes learned from high-quality annotations can effectively guide the attention-based aggregation for unlabelled branches.

#### 3.3.2. Weakly-Supervised Learning via Dynamic Prototypes

For data lacking segment-level annotations, we employ the calibrated prototypes for weakly-supervised learning. First, we compute the cosine similarity between the geometric feature vector of each segment $v_{geo}^{\left(l\right)}$ and the learned prototypes $m_{k}$ to generate a similarity score matrix $S\in R^{L\times 4}$, where $S_{l,k}=\cos\left(v_{geo}^{\left(l\right)},m_{k}\right)$. To aggregate these segment-level signals into a branch-level prediction, we employ a severity-aware attention mechanism. Recognizing that the branch-level diagnosis is typically defined by the most severe lesion, we weight each segment’s contribution based on its maximum similarity response to the prototypes. The branch-level probability distribution ${\hat{Y}}_{br}$ is computed as:

(9) $${\hat{Y}}_{br}=\frac{\sum_{l=1}^{L}\exp\left(\max_{k}S_{l,k}\right)\cdot \mathrm{Softmax}\left(S_{l,\cdot }\right)}{\sum_{j=1}^{L}\exp\left(\max_{k}S_{j,k}\right)}.$$

The weak supervision loss is then defined as follows:

(10) $$L_{weak}=\mathrm{CE}\left({\hat{Y}}_{br},y_{br}\right),$$

where $y_{br}$ denotes the ground-truth branch label.

#### 3.3.3. Logical Regularization and Joint Objective

Furthermore, to ensure clinical plausibility and maximize the utility of coarse-labelled data, we enforce logical consistency constraints across the entire dataset. A mutual exclusion loss $L_{mutex}$ is designed to penalize pathological contradictions, specifically the scenario where the network predicts significant stenosis (k>0) but simultaneously predicts the absence of plaque (Class 0). This constraint is mathematically defined as:

(11) $$L_{mutex}=\frac{1}{L}\sum_{l=1}^{L}\left[\left(\sum_{k=1}^{3}P_{st}^{\left(l,k\right)}\right)\cdot P_{pl}^{\left(l,0\right)}+P_{st}^{\left(l,0\right)}\cdot \left(1-P_{pl}^{\left(l,0\right)}\right)\right].$$

It is important to note that this penalty is unidirectional; it targets the illogical “stenosis without plaque” scenario but does not penalize “plaque without stenosis”. Quantitative analysis in Section 5.1.1 confirms that this design does not suppress the detection of non-obstructive plaques. Additionally, a hierarchical consistency loss $L_{cons}$ enforces that the aggregated risk derived from local segment predictions aligns with the global branch-level risk $P_{global}$ predicted directly by the auxiliary head. We employ a smooth-max operator to approximate the branch-level severity from segment probabilities, minimizing the mean squared error:

(12) $$L_{cons}={∥\mathrm{SmoothMax}\left(P_{st}\right)-P_{global}∥}^{2}.$$

Specifically, to ensure differentiability while capturing the “worst-case” stenosis scenario characteristic of clinical diagnosis, the SmoothMax function is formulated as a LogSumExp approximation:

(13) $$\mathrm{SmoothMax}{\left(P_{st}\right)}_{k}=\frac{1}{\alpha }\log\left(\sum_{l=1}^{L}\exp\left(\alpha \cdot P_{st}^{\left(l,k\right)}\right)\right),$$

where α is a temperature hyperparameter controlling the approximation sharpness (set to α=10), effectively forcing the global prediction to align with the most severe localized stenosis detected in the CA sequence. The final objective function dynamically balances the task-specific losses and consistency constraints using an uncertainty-based automatic weighting mechanism to ensure stable convergence:

(14) $$L_{total}=\frac{1}{2\sigma_{1}^{2}}L_{task}+\frac{1}{2\sigma_{2}^{2}}\left(L_{mutex}+L_{cons}\right)+\log\left(\sigma_{1}\sigma_{2}\right),$$

where $L_{task}$ dynamically switches between the strong supervision loss $L_{strong}$ and the weak supervision loss $L_{weak}$ depending on the availability of fine-grained annotations.

## 4. Experiment Configurations

### 4.1. Dataset

We collected CCTA images of 350 subjects from a local hospital in China (Nan Fang Hospital, the First Affiliated Hospital of Southern Medical University). Two experienced physicians annotated CA segments with plaque classification and stenosis grading. Prior to annotation, standardized labeling of the CA branches was performed following [37] and then transformed to the CPR form. We implemented a dual-granularity annotation strategy comprising fine-grained and coarse-grained protocols. The fine-grained annotation (N=200) was conducted at the segment level. In this subset, plaque composition was categorized into three types (calcified, non-calcified, and mixed), and stenosis severity was stratified into four grades: normal, mild, moderate, and severe. Conversely, the coarse-grained annotation (N=150) was performed at the branch level, recording exclusively the maximum stenosis grade observed within each entire branch. Regarding the experimental setup, we implemented a strict patient-level splitting strategy to prevent data leakage. First, we held out 20% of the total cohort (70 subjects), selected exclusively from the fine-grained subset, as an independent test set to enable rigorous pixel-level evaluation. The remaining 280 subjects (130 fine-grained + 150 coarse-grained) formed the development pool for 5-fold cross-validation. Specifically, this pool was randomly partitioned into five mutually exclusive folds at the patient level using stratified sampling to maintain a consistent ratio of fine-to-coarse annotations within each fold. In each cross-validation round, four folds served as the training set while the remaining fold functioned as the validation set to monitor convergence and optimize hyperparameters. All input images were preprocessed to extract centerlines and generate paired CPR ($X_{MPR}$) and volumetric patches ($X_{3D}$).

### 4.2. Implementation Details

We conducted all experiments in PyTorch (version 1.13.0) on an NVIDIA 3090 GPU platform (NVIDIA Corporation, Santa Clara, CA, USA). The networks were optimized via Adam (learning rate: $10^{-4}$) for 800 epochs, using a batch size of 3. Hyper-parameter tuning and model training relied solely on the training partition to ensure rigorous evaluation.

### 4.3. Comparison with State-of-the-Art Methods

To demonstrate the effectiveness and superiority of our proposed MG-HGLNet, we conducted comparative experiments with the state-of-the-art (SOTA) methods. Methods presented by Tejero-de Pablos et al. [16], Zreik et al. [10], Denzinger et al. [17], Zhang et al. (2022) [22], Van Herten et al. [14], Ma et al. [38], Zhang et al. (2025) [12] and le et al. [19] were applied for stenosis grading. Methods presented by Zreik et al. [10], Zhang et al. [22], Van Herten et al. [14] and Ma et al. [38] were applied for plaque classification. To ensure a strictly fair comparison, all baseline methods were re-trained on the identical dataset splits using a unified preprocessing framework. Input modalities were standardized: 2D methods utilized the same CPR sequences as our method, while 3D methods utilized the same volumetric patches. Regarding training configurations, we acknowledge that different architectures (e.g., CNN vs. Mamba) require distinct hyperparameter settings to achieve convergence. Therefore, strictly enforcing identical hyperparameters would be unfair and might handicap certain baselines. Instead, to ensure a fair comparison of optimal performance, we adopted an architecture-specific optimization strategy, where learning rates and optimizers were tailored to each model’s architectural requirements, and an early stopping mechanism was employed to ensure full convergence.

### 4.4. Evaluation Metrics

To comprehensively evaluate the performance of our proposed method, we employed a set of standard quantitative metrics derived from the confusion matrix. These metrics include Accuracy (Acc), Precision (Prec), Specificity (Spec), Negative Predictive Value (NPV), and the F1-score.

Let TP, TN, FP, and FN denote the number of true positives, true negatives, false positives, and false negatives, respectively. **Acc** measures the overall correctness of the model across all classes and is defined as:

(15) $$\mathrm{Acc}=\frac{TP+TN}{TP+TN+FP+FN}$$

**Prec** quantifies the proportion of positive identifications that were actually correct:

(16) $$\mathrm{Prec}=\frac{TP}{TP+FP}$$

**Spec** evaluates the model’s ability to correctly identify negative cases:

(17) $$\mathrm{Spec}=\frac{TN}{TN+FP}$$

**NPV** indicates the probability that a sample predicted as negative is actually negative:

(18) $$\mathrm{NPV}=\frac{TN}{TN+FN}$$

Finally, the **F1-score** provides a harmonic mean of Precision and Recall, offering a more balanced view of performance, especially when handling imbalanced datasets:

(19) $$F1=\frac{2TP}{2TP+FP+FN}$$

For multi-class classification tasks, these metrics are calculated for each class using a One-vs-Rest approach and then averaged using Macro-averaging to treat all classes equally.

## 5. Results and Analysis

In this section, we systematically evaluate the performance of the proposed MG-HGLNet on the in-house dataset. First, we present a comprehensive comparison with current SOTA methods for both CA stenosis grading and plaque classification tasks to demonstrate the superiority of our framework. Subsequently, we provide qualitative visualizations to intuitively analyze the model’s capability. Finally, we conduct detailed ablation studies to verify the effectiveness of the individual components.

### 5.1. Comparisons with the State-of-the-Art Methods

To demonstrate the effectiveness and superiority of our proposed MG-HGLNet, we conducted comprehensive comparative experiments against SOTA methods on the same dataset. The comparison includes established approaches for stenosis grading and plaque classification. To ensure a rigorous evaluation, all experiments were performed using 5-fold cross-validation, and the results are reported as Mean ± Standard Deviation.

#### 5.1.1. Quantitative Results

As presented in Table 1, MG-HGLNet establishes a new benchmark for stenosis grading, achieving a remarkable Acc of 92.4% and an F1 Score of 91.8%. In a comparison with the second-best method (Le et al.), our approach demonstrates consistent improvements across all evaluation metrics: 3.1% in Acc, 3.1% in Prec, 3.4% in Sens, 3.2% in Spec, and 3.3% in F1 Score. More importantly, given the clinical imperative to avoid fatal missed diagnoses in screening scenarios, our method demonstrates superior reliability with a Sens of 92.1%. This represents a statistically significant improvement over the leading Transformer-based methods of Le et al. (3.4%) and Ma et al. (4.7%). This high Sens is directly attributed to our TDE module utilizing BiV-Mamba. Unlike previous approaches that may struggle with the continuous nature of CA tapering, our model captures the global context from the proximal to the distal CA. This global modeling allows for accurate calibration of the luminal diameter, thereby reducing false negatives that often occur with methods limited by local receptive fields.

For plaque classification, Table 2 illustrates that MG-HGLNet achieves state-of-the-art results with an Acc of 91.5%. In the context of CCTA analysis, high Spec and Prec are critical to mitigating the calcium blooming artifact, which frequently leads to the misclassification of calcified plaques as mixed or soft plaques in conventional models. Our method effectively addresses this bottleneck, achieving a Spec of 94.2% and a Prec of 90.9%. Compared to the strongest baseline method (Van Herten et al.), which integrates mesh priors, our model improves Spec by 4.8% and Prec by 5.5%. This performance gain validates the effectiveness of our SSD module. By employing frequency-domain analysis to extract unique texture fingerprints and explicitly disentangling them from geometric boundary features, MG-HGLNet avoids the feature coupling issues prevalent in previous works, thereby providing a precise characterization of complex plaque compositions that aligns closely with expert consensus. To verify that the mutual exclusion constraint does not hinder early lesion detection, we evaluated the model on the non-obstructive plaque subgroup (stenosis ≤ mild). The model achieved a Sens of 89.4%, comparable to the 92.8% Sens on obstructive plaques, confirming robust detection of early-stage atherosclerosis

#### 5.1.2. Qualitative Results

**Figure 3:**
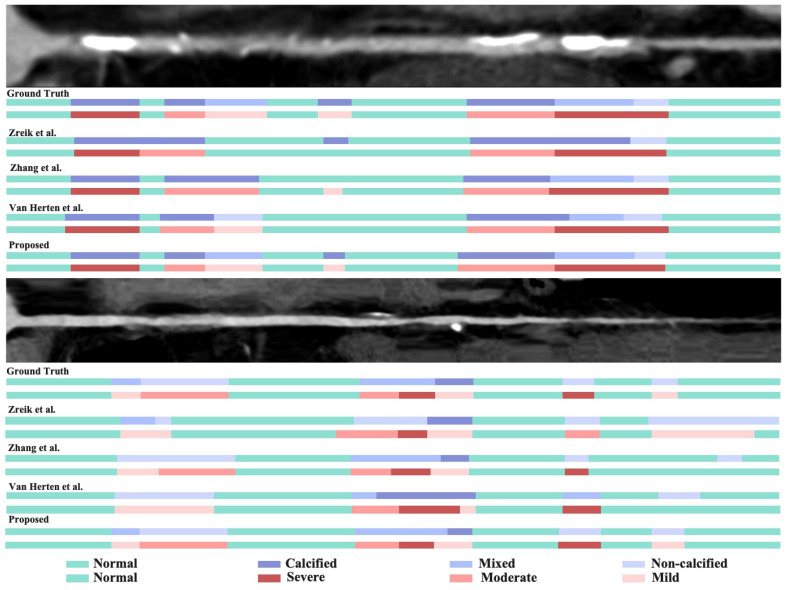
Qualitative visualization of CAD analysis. The color-coded masks represent plaque types (Normal, Calcified, Mixed, Non-calcified) and stenosis grades (Normal, Severe, Moderate, Mild).

The visualization results of plaque classification and stenosis grading are shown in Figure 3, illustrating the powerful visual superiority of the proposed MG-HGLNet on both tasks. As illustrated in the top panel of Figure 3, we present a challenging case characterized by dense calcified plaques and severe luminal stenosis. A detailed visual inspection reveals a twofold limitation in the comparative methods. First, these methods exhibit a distinct lack of sensitivity to subtle, focal lesions. Second, for the dominant severe stenosis, they suffer from significant spatial over-estimation. The predicted lesion boundaries are coarsely dilated beyond the ground truth, due to the interference of calcium artifacts which mask the true CA lumen. In contrast, the MG-HGLNet effectively alleviates this trade-off between sensitivity and precision. It not only accurately delineates the boundaries of the severe stenosis without unnecessary dilation but also successfully detects the smaller, discrete plaque/stenosis fragments that are missed by the comparative methods. It demonstrates that our MG-HGLNet can effectively reduce the impact of calcification artifacts while maintaining high sensitivity to subtle texture changes within smaller lesions. The bottom panel highlights the model’s robustness in alleviating false positives. While competing methods erroneously predict physiological tapering in the proximal segments as “mild” or “moderate” stenosis, the MG-HGLNet effectively suppresses these disturbances. This superior specificity is attributed to the TDE module’s ability to model global topological dependencies, enabling the network to distinguish between pathological narrowing and normal CA lumen variation.

### 5.2. Ablation Study

#### 5.2.1. Quantitative Comparison of Ablation Study

To investigate the individual contribution of each component in MG-HGLNet, we conducted an ablation study. We established a baseline model consisting of a standard Mamba encoder without the bidirectional mechanism, decoupling mechanism, and mixed-grained supervision. We then incrementally integrated the TDE module, SSD module, and MSO module.

Table 3 details the step-wise performance improvements across all evaluation metrics. The integration of the TDE module yielded the most significant improvement in Sens for stenosis grading, which surged from 85.3% (Baseline) to 88.9% (+3.6%). The baseline model, lacking global topological awareness, frequently misidentified physiological tapering as mild stenosis, resulting in a lower sensitivity. The TDE module effectively establishes the global topological by modeling the proximal-to-distal hemodynamic context. This allows the network to distinguish true pathological narrowing from normal vessel morphology, drastically reducing missed diagnoses in early-stage lesions. The addition of the SSD module resulted in a crucial boost in Spec for plaque classification, elevating it from 89.1% to 92.4% (+3.3%). In the baseline and TDE-only models, the Spec was limited by calcium blooming artifacts and surrounding similar tissue, leading to false positives (i.e., classifying calcified plaques as “Mixed”). The SSD module explicitly decouples the spectral texture features from geometric boundary features. This disentanglement ensures that artifacts do not contaminate the morphological assessment, thereby significantly improving the model’s ability to reject false positives. By leveraging the large volume of coarse-grained branch labels via Anatomy-Aware Dynamic Prototypes, the MSO strategy enhanced the model’s generalization capability. This proved particularly effective for “hard examples” (e.g., intermediate stenosis or small mixed plaques), resulting in a comprehensive improvement of 1.6% Sens and 1.4% F1 in stenosis grading and 1.8% Spec and 2.1% F1.

#### 5.2.2. Qualitative Comparison of Ablation Study

To further validate the effectiveness of the proposed modules and the robustness of our semi-supervised strategy, we conducted experiments in terms of visualization.

**Figure 4:**
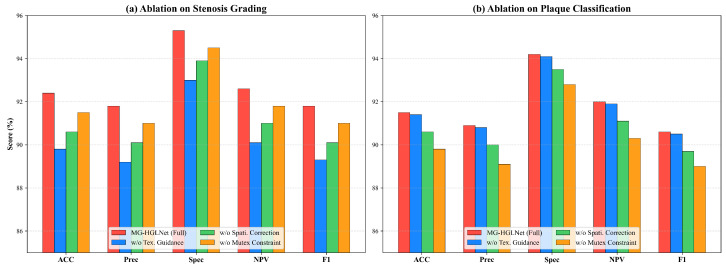
Component-wise ablation study on the test set across five evaluation metrics (ACC, Prec, Spec, NPV, and F1). (**a**) Impact on Stenosis Grading: The removal of Texture Guidance results in the most significant performance drop, confirming that texture features are critical for defining luminal boundaries in soft plaques. (**b**) Impact on Plaque Classification: The removal of the Mutex Constraint causes the largest decline, highlighting the importance of logical consistency in reducing false positives. Notably, removing Texture Guidance has negligible impact on plaque classification as the texture branch itself remains intact.

As shown in Figure 4, to dissect the specific contribution of each component within the MG-HGLNet, we evaluated the full model against three variants: “w/o Texture Guidance”, which removes the feature injection pathway in the SSD module where spectral texture features ($F_{tex}$) guide geometric attention; “w/o Geo. Correction”, which eliminates the attention-based rectification in the TDE module; and “w/o Mutex Constraint”, which removes the logical mutual exclusion loss ($L_{mutex}$) from the MSO strategy.For stenosis grading (Figure 4a), the results reveal a distinct hierarchy of feature dependencies where the “w/o Texture Guidance” variant exhibits the most severe performance degradation across all metrics (Acc drops to 89.8%). This critical finding indicates that accurate stenosis quantification is not purely a geometric task; for non-calcified plaques with low contrast, the geometric branch heavily relies on semantic cues provided by the texture branch to define precise luminal boundaries. Following this, the “w/o Geo. Correction” variant shows the second-largest drop, confirming that while deep networks possess some translational invariance, correcting spatial distortions inherent in CPR is still essential for reliable diameter quantification in tortuous CA. The performance dynamics shift significantly for plaque classification (Figure 4b). In contrast to stenosis grading, the “w/o Mutex Constraint” variant results in the lowest performance here (Acc 89.8%), highlighting that logical consistency acts as a powerful regularizer to suppress contradictory false positives (e.g., noise mimicking calcification) that do not correlate with geometric narrowing. Conversely, the “w/o Texture Guidance” variant maintains performance nearly identical to the Full Model (Acc 91.4%). This validates our decoupling hypothesis: since the texture branch itself remains intact, it is fully capable of extracting spectral fingerprints for characterization independently, demonstrating that the guidance flow is beneficial to the geometric receiver without compromising the texture sender.

**Figure 5:**
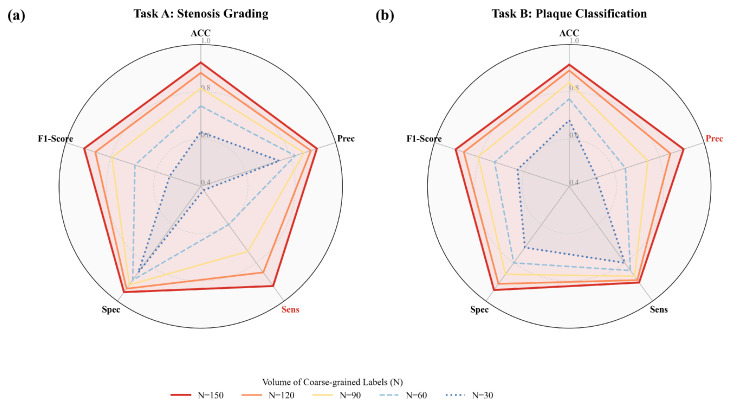
Radar charts illustrating the performance metrics of (**a**) Stenosis Grading and (**b**) Plaque Classification across varying numbers of coarse-grained labeled data.

To assess the practical value of the MSO strategy, we evaluated the model’s performance when trained with decreasing volumes of coarse-grained branch labels (N={150,120,90,60,30}), while keeping fine-grained segment labels constant. As illustrated in Figure 5, the results not only confirm the label efficiency of MG-HGLNet but also reveal a significant heterogeneity in how supervision granularity impacts different diagnostic mechanisms. First, MG-HGLNet demonstrates superior robustness, surpassing some existing methods even under lower proportion of coarse-grained labels. Even with only 20% of the coarse-grained labels (N=30), the model establishes a high performance floor: for stenosis grading, it maintains a sensitivity of 84.0%, outperforming the 83.6% reported by Zreik et al. [10]; for plaque classification, it sustains a precision of 86.0%, exceeding the 85.4% benchmark of Van Herten et al. [14]. This validates that our weakly-supervision framework relies on not only the data volume but also the data utilization efficiency. Second, as shown in Figure 5a, as coarse-grained labels decrease, the stenosis grading task exhibits a characteristic “Sensitivity Collapse,” where sensitivity declines from 92.1% to 84.0%, representing the most significant impact. This phenomenon indicates that without sufficient branch-level annotations to anchor the Anatomy-Aware Dynamic Prototypes, the model struggles to establish long-range semantic consistency across the CA tree. When validating intermediate or ambiguous lesions, the network loses confidence in assigning high-severity grades, retreating to a conservative “high-specificity, low-sensitivity” prediction mode, thereby increasing the rate of missed diagnoses. As shown in Figure 5b, the influence of decreasing coarse-grained supervision on plaque classification warrants specific attention, given that these annotations exclusively characterize luminal stenosis severity rather than plaque composition. Despite this indirect supervision, we observe a “Precision Collapse” (precision drops by 4.9%) while sensitivity remains stable. This confirms that branch-level stenosis labels provide a critical “Pathological Context” for plaque detection. Specifically, the Logical Mutual Exclusion Constraint leverages these labels to enforce clinical consistency: if a branch is globally labeled as “Normal” (no stenosis), the model learns to suppress localized false positives caused by imaging artifacts (e.g., calcium blooming or noise) that mimic plaque textures. As this global guidance diminishes (N=150→30), the model loses its ability to confidently rule out artifacts in non-stenotic vessels, leading to an aggressive recall strategy that maintains sensitivity but suffers from increased false positives (lower precision).

In summary, while MG-HGLNet maintains a clinically acceptable performance even with minimal coarse-grained labeled data, this analysis underscores that abundant coarse-grained data remains indispensable for minimizing missed diagnoses in stenosis grading and mitigating false positives in plaque characterization.

## 6. Discussion

**Figure 6:**
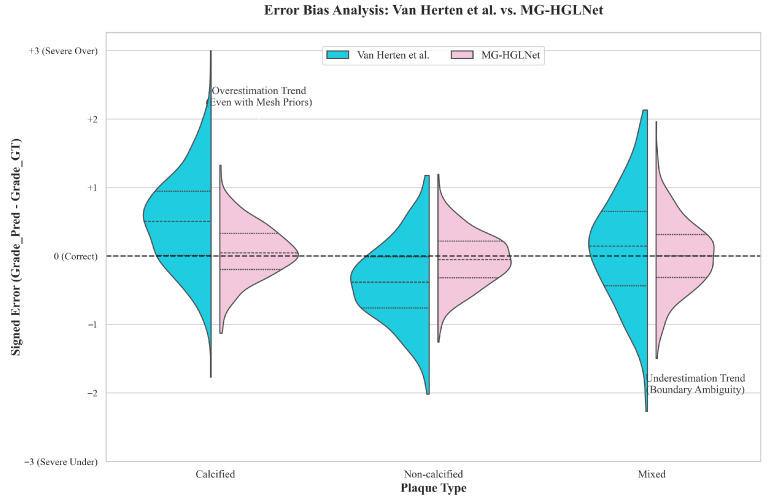
Distribution of signed stenosis grading errors ($E=Grade_{Pred}-Grade_{GT}$) stratified by plaque type. The split violin plots visualize the systematic bias and variance between the previous method (Van Herten et al., blue) and the proposed MG-HGLNet (pink). The horizontal dashed line at y=0 denotes unbiased prediction.

To thoroughly investigate the impact of plaque composition on stenosis grading, we analyze the signed error distribution across three subgroups as illustrated in Figure 6. The comparative results reveal distinct biases in the previous method (Van Herten et al.) that are effectively rectified by our proposed MG-HGLNet. Specifically, in the calcified plaque group, the previous method exhibits a significant positive skewness (density mass concentrated above y=0), indicating a tendency towards overestimation of stenosis grading. Conversely, for non-calcified plaques, the previous method displays a notable negative shift (density mass below y=0), reflecting a propensity for underestimation or missed diagnosis. In the heterogeneous mixed plaque group, while the directional bias is less pronounced, the previous method suffers from a broad dispersion with high variance. In contrast, the MG-HGLNet maintains a tight, zero-centered distribution across all subgroups, demonstrating that our method achieves robust quantification regardless of plaque complexity.

The observed rectification of these biases provides direct evidence for the synergistic effectiveness of the modules within the MG-HGLNet. The elimination of positive bias in calcified lesions is attributed to the SSD module. By employing FB-Mamba to extract spectral fingerprints, the SSD module explicitly disentangles high-frequency calcification signals from geometric features, preventing artifacts from being misinterpreted as luminal narrowing. Furthermore, the correction of negative bias in non-calcified lesions relies on the TG-DBA, which is a core sub-component of the SSD module. It leverages decoupled texture semantics as a prior to guide deformable sampling, thereby recovering precise stenosis boundaries even in low-contrast scenarios. Furtherly, the overall robustness and false estimation are further improved by the BiV-Mamba. By modeling long-range dependencies bidirectionally along the CA, BiV-Mamba establishes a global anatomical context, allowing the network to distinguish true pathological stenosis from physiological tapering, which is essential for correcting grading errors in subtle or complex lesions.

**Figure 7:**
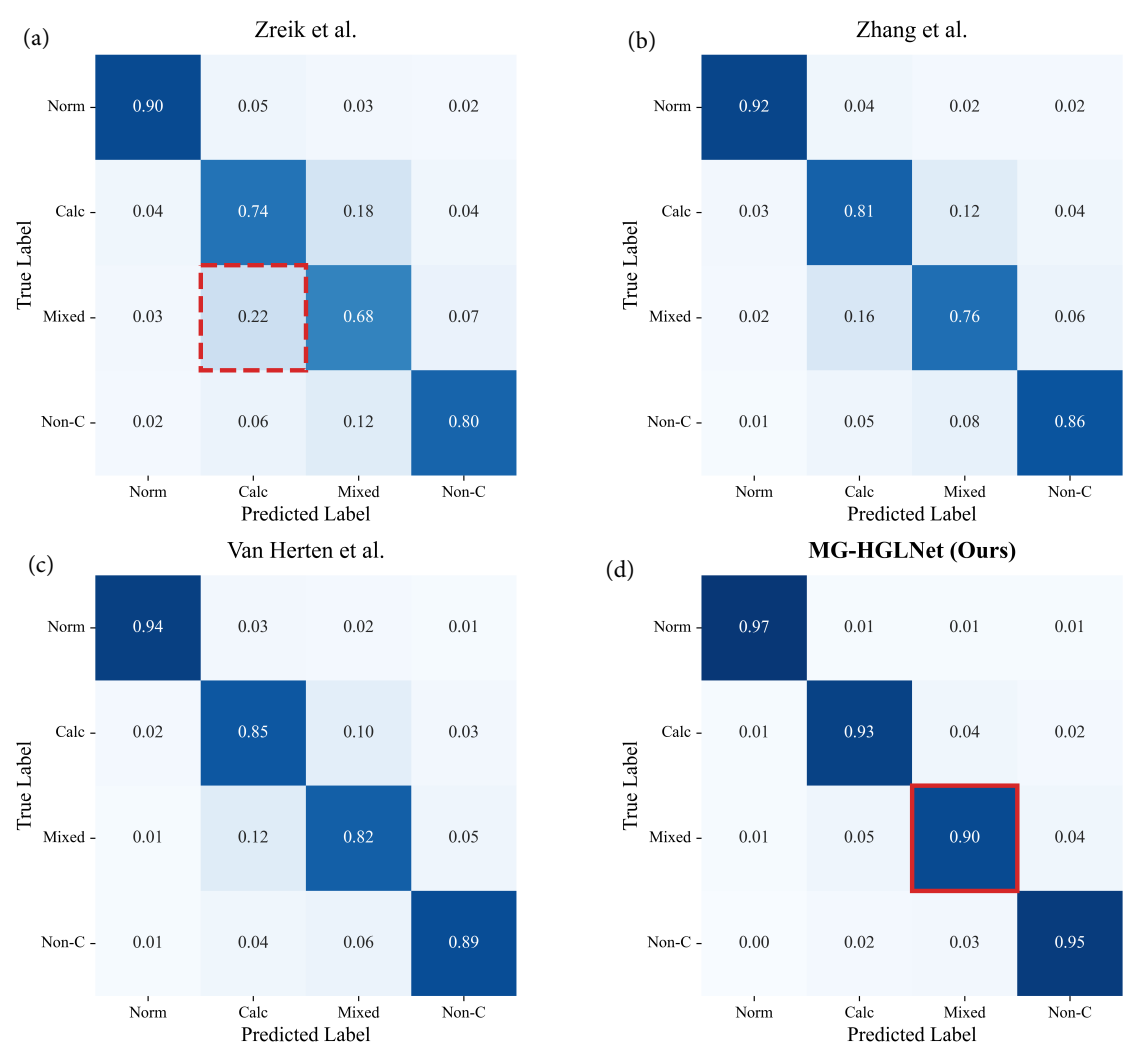
Confusion matrices of plaque classification comparisons. The matrices visualize the classification performance of (**a**) Zreik et al., (**b**) Zhang et al., (**c**) Van Herten et al., and (**d**) the proposed MG-HGLNet.

To provide a granular analysis of the classification performance, Figure 7 presents the confusion matrices for the comparative methods. As illustrated in Figure 7a–c, existing SOTA methods exhibit a deficiency in identifying mixed plaques, frequently misclassifying them as calcified plaques. Specifically, Zreik et al. and Zhang et al. show misclassification rates of 22% and 16%, respectively. This phenomenon is attributed to “calcium blooming,” where the high-intensity signals from calcified components in mixed plaques undergo spatial expansion during CT reconstruction. These dominant high-frequency artifacts tend to mask the subtle, low-contrast textural features of the co-existing lipid or fibrous components, causing competitive methods to categorize the entire plaque as “calcified”. In contrast, the proposed MG-HGLNet (Figure 7d) effectively mitigates this feature coupling issue. Our method achieves a classification accuracy of 90% for mixed plaques (red solid box), reducing the misclassification rate into the calcified category to 5%. This phenomenon reflects the robustness of our MG-HGLNet for heterogeneous plaque classification.

**Figure 8:**
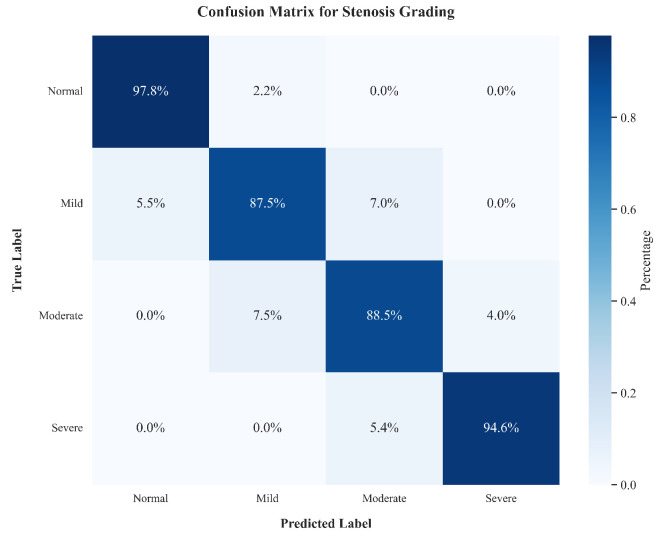
Confusion matrix for stenosis grading on the test set. The matrix illustrates that misclassifications primarily occur between adjacent grades (e.g., Mild vs. Moderate), while the model maintains high accuracy (94.6%) for critical Severe cases.

To further scrutinize the clinical reliability of MG-HGLNet, we analyzed the per-class performance for stenosis grading, as presented in Table 4 and Figure 8. The model exhibits exceptional Senc for Severe stenosis (94.6%) and Normal segments (97.8%). This polar distribution of performance is clinically favorable, as it minimizes the risk of missing high-risk interventional candidates (severe cases) while effectively ruling out healthy subjects to avoid overtreatment. Conversely, a slight dip in performance is observed for Mild and Moderate grades (F1-scores of 88.3% and 88.0%, respectively). As visualized in the confusion matrix (Figure 8), errors in these categories are predominantly confined to adjacent grades (e.g., Mild misclassified as Moderate). This pattern mirrors the inherent inter-observer variability often seen in clinical practice, where the boundary between Mild and Moderate stenosis can be ambiguous even for experienced radiologists. Nevertheless, the high Spec across all classes (>93%) confirms that the model’s predictions remain robust and trustworthy.

To analyze the efficiency of our MG-HGLNet, we evaluated the computational efficiency on an NVIDIA RTX 3090. The proposed MG-HGLNet contains 42.5 M parameters and operates with 12.8 GFLOPs. The inference time is approximately 85 ms per patient. The preprocessing stage (automated centerline extraction and CPR generation) requires approximately 4.5 s per patient, which fits within the clinically acceptable timeframe for CCTA analysis.

## 7. Conclusions

In this study, we presented MG-HGLNet, an end-to-end framework designed for the comprehensive assessment of CA lesions from CCTA images. By integrating the TDE module, we effectively modeled the continuous anatomical topology and mitigated geometric distortions, thereby enhancing the sensitivity to stenosis in tortuous CAs. The proposed SSD module successfully addressed the challenge of feature entanglement by synergizing spectral texture analysis with morphological attention, which significantly improved the specificity of plaque characterization, particularly in the presence of calcium blooming artifacts. Furthermore, the MSO strategy demonstrated that integrating anatomy-aware dynamic prototypes with logical constraints allows for the efficient utilization of coarse-grained branch labels, bridging the gap between data availability and annotation costs. The proposed framework is designed to alleviate the burden of pixel-level annotation, making it highly adaptable to clinical datasets where only report-level labels are available. In terms of deployment, while the inference time is real-time, the preprocessing pipeline remains a bottleneck. Future integration into clinical PACS systems will require optimizing the centerline extraction step.

Despite the promising results, this study has several limitations that warrant further investigation. First, the primary limitation of this study is the use of a single-center dataset (*N* = 350). While the model demonstrates strong internal validity, its performance across different scanner vendors (e.g., GE, Siemens, Philips) and reconstruction kernels remains to be verified. Variations in image noise and spatial resolution could impact the efficacy of the SSD module. Second, the ground truth relies on expert anatomical assessment rather than invasive functional metrics like Fractional Flow Reserve (FFR). While our system effectively automates the CAD-RADS classification workflow, it does not directly predict hemodynamic significance. Future work will focus on two main directions: (1) collecting multi-center data to evaluate the model’s robustness against domain shifts; and (2) integrating functional assessment into the MG-HGLNet framework alongside anatomical stenosis. 

## Figures and Tables

**Table 1:** Quantitative comparison of **coronary artery stenosis grading** (4-class) on the test set. Best results are highlighted in **bold**. Values are presented as Mean ± Std (%).

Methods	Acc	Prec	Sens	Spec	F1 Score
Tejero-de Pablos et al. [16]	82.1 ± 1.9	81.3 ± 2.4	80.4 ± 2.1	85.2 ± 1.5	80.7 ± 2.0
Denzinger et al. [17]	83.7 ± 1.4	82.5 ± 1.6	82.1 ± 1.8	86.4 ± 1.2	82.3 ± 1.5
Zreik et al. [10]	85.3 ± 1.2	84.4 ± 1.4	83.6 ± 1.3	88.1 ± 1.0	83.9 ± 1.3
Zhang et al. [22]	86.9 ± 1.1	85.7 ± 1.0	85.4 ± 1.1	89.6 ± 0.9	85.5 ± 1.1
Van Herten et al. [14]	87.6 ± 0.8	86.4 ± 0.9	86.1 ± 1.0	90.3 ± 0.7	86.2 ± 0.8
Ma et al. [38]	88.1 ± 0.9	87.3 ± 0.8	87.4 ± 0.9	91.1 ± 0.6	87.1 ± 0.8
Le et al. [19]	89.3 ± 0.7	88.6 ± 0.7	88.7 ± 0.8	92.1 ± 0.5	88.5 ± 0.7
Zhang et al. [12]	87.9±0.9	86.8±1.0	86.5±0.9	90.5±0.8	86.9±0.9
**MG-HGLNet (Ours)**	**92.4 ± 0.5**	**91.7 ± 0.6**	**92.1 ± 0.6**	**95.3 ± 0.4**	**91.8 ± 0.5**

**Table 2:** Quantitative comparison of **plaque classification** (4-class: Normal, Calcified, Mixed, Non-calcified) on the test set. Best results are highlighted in **bold**. Values are presented as Mean ± Std (%).

Methods	Acc	Prec	Sens	Spec	F1 Score
Zreik et al. [10]	80.4 ± 1.7	79.1 ± 1.9	78.6 ± 2.0	84.3 ± 1.4	78.7 ± 1.8
Zhang et al. [22]	84.5 ± 1.3	83.2 ± 1.5	82.9 ± 1.4	87.6 ± 1.1	83.0 ± 1.3
Van Herten et al. [14]	86.3 ± 0.9	85.4 ± 1.0	85.1 ± 1.1	89.4 ± 0.8	85.2 ± 0.9
Ma et al. [38]	89.5±0.7	88.6±0.8	88.2±0.9	92.1±0.6	88.4±0.7
**MG-HGLNet (Ours)**	**91.5 ± 0.6**	**90.9 ± 0.7**	**90.3 ± 0.7**	**94.2 ± 0.5**	**90.6 ± 0.6**

**Table 3:** Ablation study of the proposed modules. All evaluation metrics are reported as percentages (%). **TDE**: Topology-Aware Dual-Stream Encoding; **SSD**: Synergistic Spectral–Morphological Decoupling; **MSO**: Mixed-Grained Supervision Optimization.

Modules			Stenosis Grading Task					Plaque Classification Task				
TDE	SSD	MSO	Acc	Prec	Sens	Spec	F1	Acc	Prec	Sens	Spec	F1
−	−	−	86.5	85.8	85.3	88.0	85.6	85.2	84.5	83.1	87.8	83.8
✓	−	−	89.1	88.4	88.9	90.5	88.6	86.8	86.1	85.4	89.1	85.7
✓	✓	−	90.8	90.2	90.5	93.1	90.4	89.8	89.0	88.7	92.4	88.5
✓	✓	✓	92.4	91.7	92.1	95.3	91.8	91.5	90.9	90.3	94.2	90.6

**Table 4:** Per-class performance metrics for stenosis grading on the test set.

Class	Prec	Sens	Spec	F1
Normal	96.2	97.8	97.9	97.0
Mild	89.1	87.5	93.8	88.3
Moderate	87.5	88.5	93.0	88.0
Severe	94.0	94.6	96.5	94.3
Macro-Avg	91.7	92.1	95.3	91.8

## Data Availability

The data presented in this study are available on request from the corresponding author. Data are not publicly available due to privacy and ethical reasons.

## Abbreviations

The following abbreviations are used in this manuscript:

BiV-Mamba	Bidirectional Vessel Mamba
CA	Coronary Artery
CAD	Coronary Artery Disease
CAD-RADS	Coronary Artery Disease-Reporting and Data System
CAMs	Class Activation Maps
CCTA	Coronary Computed Tomography Angiography
CE	Cross-Entropy
CNN	Convolutional Neural Network
CPR	Curved Planar Reformation
DL	Deep Learning
EMA	Exponential Moving Average
FB-Mamba	Frequency-Band Mamba
FFT	Fast Fourier Transform
GAP	Global Average Pooling
GCN	Graph Convolutional Network
MG-HGLNet	Mixed-Grained Hierarchical Geometric-Semantic Learning Network
MLP	Multi-Layer Perceptron
MPR	Multi-Planar Reformatted
MSO	Mixed-Grained Supervision Optimization
NPV	Negative Predictive Value
RCNN	Recurrent Convolutional Neural Network
SAM	Spatial-Aware Cross-Attention Module
SSD	Synergistic Spectral–Morphological Decoupling
SSM	Structured State Space Model
TDE	Topology-Aware Dual-Stream Encoding
TG-DBA	Texture-Guided Deformable Boundary Attention
ViTs	Vision Transformers
VSS	Visual State Space
